# Efficacy of Polyvalent Human Immunoglobulins in an Animal Model of Neuromyelitis Optica Evoked by Intrathecal Anti-Aquaporin 4 Antibodies

**DOI:** 10.3390/ijms17091407

**Published:** 2016-08-26

**Authors:** Benedikt Grünewald, Jeffrey L. Bennett, Klaus V. Toyka, Claudia Sommer, Christian Geis

**Affiliations:** 1 Hans-Berger Department of Neurology, Jena University Hospital, Am Klinikum 1, 07747 Jena, Germany; christian.geis@med.uni-jena.de; 2 Integrated Research and Treatment Center—Center for Sepsis Control and Care (CSCC), Jena University Hospital, Am Klinikum 1, 07747 Jena, Germany; 3 Department of Neurology, University Hospital Würzburg, Josef-Schneider-Straße 11, 97080 Würzburg, Germany; kv.toyka@uni-wuerzburg.de (K.V.T.); sommer@uni-wuerzburg.de (C.S.); 4 Departments of Neurology and Ophthalmology, University of Colorado Denver, Aurora, CO 80045, USA; Jeffrey.Bennett@ucdenver.edu

**Keywords:** NMOSD, aquaporin 4, autoantibody, IVIg, intrathecal application

## Abstract

Neuromyelitis Optica Spectrum Disorders (NMOSD) are associated with autoantibodies (ABs) targeting the astrocytic aquaporin-4 water channels (AQP4-ABs). These ABs have a direct pathogenic role by initiating a variety of immunological and inflammatory processes in the course of disease. In a recently-established animal model, chronic intrathecal passive-transfer of immunoglobulin G from NMOSD patients (NMO-IgG), or of recombinant human AQP4-ABs (rAB-AQP4), provided evidence for complementary and immune-cell independent effects of AQP4-ABs. Utilizing this animal model, we here tested the effects of systemically and intrathecally applied pooled human immunoglobulins (IVIg) using a preventive and a therapeutic paradigm. In NMO-IgG animals, prophylactic application of systemic IVIg led to a reduced median disease score of 2.4 on a 0–10 scale, in comparison to 4.1 with sham treatment. Therapeutic IVIg, applied systemically after the 10th intrathecal NMO-IgG injection, significantly reduced the disease score by 0.8. Intrathecal IVIg application induced a beneficial effect in animals with NMO-IgG (median score IVIg 1.6 vs. sham 3.7) or with rAB-AQP4 (median score IVIg 2.0 vs. sham 3.7). We here provide evidence that treatment with IVIg ameliorates disease symptoms in this passive-transfer model, in analogy to former studies investigating passive-transfer animal models of other antibody-mediated disorders.

## 1. Introduction

Neuromyelitis Optica Spectrum Disorders (NMOSD) are a group of autoimmune diseases of the central nervous system (CNS), clinically characterized by optic neuritis and spinal cord myelitis [1]. In 70%–90% of cases, disease-specific autoantibodies to aquaporin-4 (AQP4-ABs) are detectable [2,3,4]. This discovery led to the definition that NMOSD is a distinct entity, different from multiple sclerosis [5,6]. AQP-4 is an osmotic driven water channel, highly expressed in astrocytic feet processes covering the abluminal side of capillaries, thus, contributing to the integrity of the blood-brain barrier. Binding of autoantibodies (ABs), activation of complement and cellular immune responses, collectively, are thought to be key steps in the pathogenesis of NMOSD, leading to extensive destructive lesions in the CNS grey matter, including astrocyte death [4,7,8]. Recently, we have shown in an animal model with intrathecal (i.th.) passive transfer of specific human AQP4-ABs or purified IgG fractions from NMOSD patients (NMO-IgG), that ABs can induce signs of myelopathy and cause a reduction of astrocyte surface expression of AQP4. This effect was independent of cell-infiltration and complement-activation. These findings corroborated evidence from co-cultures of astrocytes and oligodendrocytes, indicating an intrinsic pathogenic potential of AQP4-ABs [4,9,10].

The immunopathogenesis in NMOSD is now widely accepted, based on human pathology and on autoimmune animal models [8,11]. Current treatments are largely empirical and include systemic pulses of corticosteroids, plasma exchange or immunoadsorption, and immunosuppressive drugs or specific B-cell depletion using monoclonal antibodies, e.g., rituximab [12]. In small case series with NMOSD patients, pooled human immunoglobulins (IVIg) had positive effects on acute attacks and for the prevention of relapses [13,14]. Moreover, first experimental data suggest a potential positive effect of IVIg in the treatment of NMOSD: IVIg given prior to the injection of pooled purified NMO-IgG reduced disease activity in a model of NMO involving complement activation and cell infiltration [15].

IVIg has several immunomodulatory mechanisms, including direct effects on ABs, on complement activation, on T and B cell activation, suppression of AB production, and immune cell migration [16]. To elucidate the potential mechanisms of action, we here focused on the effects of IVIg on the intrinsic pathogenicity of AQP4-ABs in a defined model system, which does not show the full spectrum of immune mediators seen in other disease models of NMOSD [7,8]. We used our previously-established animal model of repetitive i.th. injections of NMO-IgG or of human monoclonal recombinant antibodies (rAB-AQP4) to study the effects of IVIg in different therapeutic paradigms. 

## 2. Results

**Figure 1:**
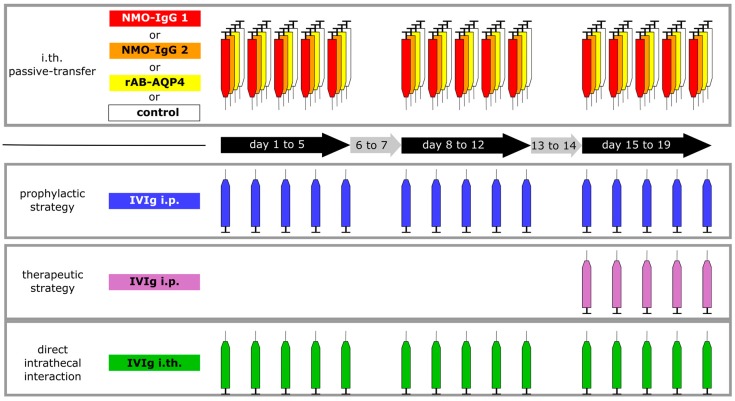
Schematic illustration of the study design. Repetitive intrathecal (i.th) application of purified patient IgG from two different patients (red and orange), recombinant human AQP4-ABs (rAB-AQP4, yellow), or control IgG (white), were performed in 3 series of five daily applications and 2 two-day breaks in between (3 weeks injection period in total). In a preventive, systemic strategy, pooled human immunoglobulin (IVIg) was applied intraperitoneally (i.p., blue). To test the therapeutic effect of IVIg in our model, IVIg was applied systemically only during the third series of i.th. passive transfer (purple). In a third approach, testing for direct antagonizing effects, IVIg was co-administered from the beginning of the experiments in an i.th. regime (green) immediately following injection of the pathogenic NMO immunoglobulin G fraction (NMO-IgG) or rAB-AQP4.

We set up a series of experiments, inducing a mild myelopathy through i.th. injection of the pathogenic NMO-IgG containing anti-AQP4-ABs. Therapeutic human IVIg was given to rats at different time points of the passive transfer, either co-administered intraperitoneally (i.p.) from onset of passive transfer (preventive strategy) or after the 10th i.th. injection at the height of the experimental disease (therapeutic strategy) (Figure 1). We used purified IgG from two different NMOSD patients or specific recombinant human AQP4-ABs (rAB-AQP4; Figure 1). As a third strategy, we tested the efficacy of IVIg when co-administrated with i.th., immediately following i.th. application of pathogenic NMO-IgG.

### 2.1. Systemic Treatment with Pooled Human Immunoglobulins (IVIg) Reduces Disease Progression in the NMO Immunoglobulin G (NMO-IgG) Passive-Transfer Model 

**Figure 2:**
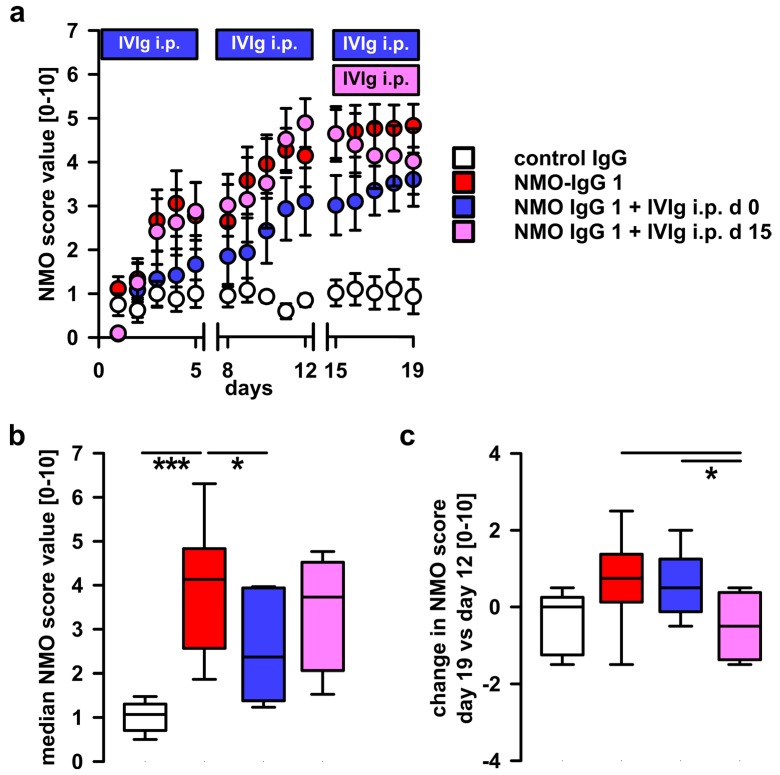
Effects of preventive and therapeutic systemic injections of IVIg. (**a**) Repetitive i.th. injections of NMO-IgG 1 led to development of disease symptoms (NMO score). The NMO-score of the NMO-IgG injected animals (red circles) increased over the experimental period and reached a plateau phase at the end of i.th. injections. The progression of the disease was slowed by concurrent i.p. application of IVIg (blue circles). Rats of the therapeutic IVIg group, receiving IVIg i.p. later, i.e., starting at day 15 (purple circles), developed comparable disease symptoms as the animals of NMO-IgG 1 (red) in before IVIg was given. After starting the IVIg injections on day 15, the NMO-score decreases over the 5 following injection days. Control IgG injected animals showed only minor abnormalities from the start (white circles). Breaks on the X-axis indicate 2 day-pauses of NMO-IgG injections. Two-way ANOVA with Bonferroni post hoc tests revealed significant differences of all groups vs. the group receiving control IgG (*p* < 0.001) and the group of NMO 1 vs. NMO1 + IVIg i.p. day 0 (*p* < 0.001); (**b**) the median NMO-score determined from the score values over the entire experiment were significantly lower when i.p. injections of IVIg were given prophylactically (one-way ANOVA, Bonferroni post hoc test; * *p* < 0.05; *** *p* ≤ 0.001; plots show median ± 25th and 75th percentiles with whiskers of the 5th and 95th percentiles); (**c**) the treatment group receiving IVIg in the therapeutic paradigm from day 15 showed a reduction of the NMO score beginning with the 11th injection while the NMO score still slightly increases in the untreated and the prophylactic treatment group but there it remained at a lower final score level (one-way ANOVA Bonferroni post hoc test, * *p* < 0.05; plots show median ± 25th and 75th percentiles with whiskers of the 5th and 95th percentiles).

Repetitive i.th. applications of the two patient IgG fractions, NMO-IgG 1 and NMO-IgG 2, led to progressive disease symptoms in rats, starting with motor abnormalities and unilateral hindlimb weakness, up to paraplegia in some severely afflicted rats, thus confirming our previous reports with different NMO-IgG preparations (Figure 2a) [10]. Rats injected with control IgG showed no abnormalities or only minor effects. In the first preventive treatment experiment, IVIg was administered i.p. from the beginning of the i.th. passive-transfer injection series of NMO-IgG. IVIg treatment led to a delay of severe disease signs and a lower disease score at the end of the experiment (Figure 2a). The disease score over the whole period of the IVIg-treated group was significantly reduced when compared to the NMO-IgG injected group without concomitant IVIg application (Figure 2b).

### 2.2. Therapeutic Potential of Systemic IVIg on Disease Progression 

To test whether systemic administration of IVIg also has the potential to reverse already existing disease symptoms in our rat model, i.p. IVIg was started only at day 15, at the time of the 11th injection of NMO-IgG. At this time point, most of the animals had developed intermediate disease signs (Figure 2a). After the initiation of IVIg-treatment, no further progression, but, rather, amelioration of disease symptoms was observed (Figure 2c). The overall disease score of animals treated using the prophylactic strategy (IVIg from day 0, Figure 2b) was lower than that of animals of the delayed therapeutic strategy group, suggesting an additional beneficial effect of IVIg in the preventive experiment during the time of NMO-IgG application (Figure 2b). When compared to the NMO scores at the time before initiation of therapeutic IVIg application from day 12, the NMO score was lower after therapeutic IVIg application on day 19 (Figure 2c). The final score values at day 19 of both treatment groups were reduced in comparison to the group receiving NMO-IgG and sham treatment (Figure 2a). 

### 2.3. Intrathecal Co-Administration of IVIg Is Effective in Reducing the Pathogenic Effects of NMO-IgG and of Recombinant Human Autoantibodies to Aquaporin-4 (rAB-AQP4)

**Figure 3:**
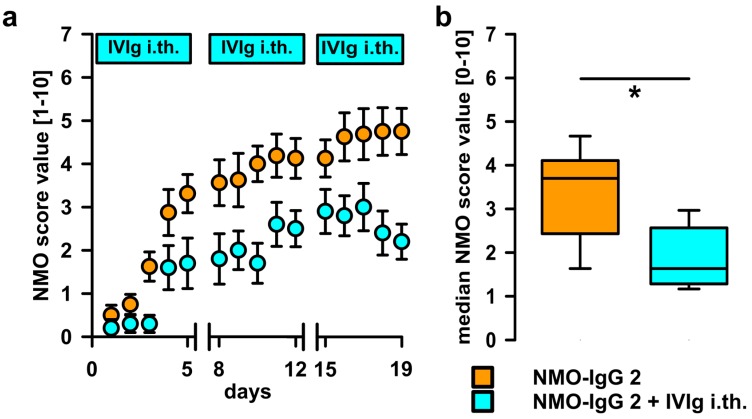
i.th. application of IVIg attenuates NMO-IgG 2 induced disease symptoms. (**a**) IVIg co-administered i.th. from day 1 of i.th. application of NMO-IgG delayed the onset and reduced the severity of disease symptoms (Two-way ANOVA with Bonferroni post hoc test *p* < 0.001 for group comparison). Breaks on the X-axis indicate 2 day-pauses of NMO-IgG; (**b**) the median disease score over the entire experiment was significantly smaller in the animals treated with i.th. IVIg (Mann-Whitney *U* test, * *p* ≤ 0.05; plots show median ± 25th and 75th percentiles with whiskers of the 5th and 95th percentiles).

**Figure 4:**
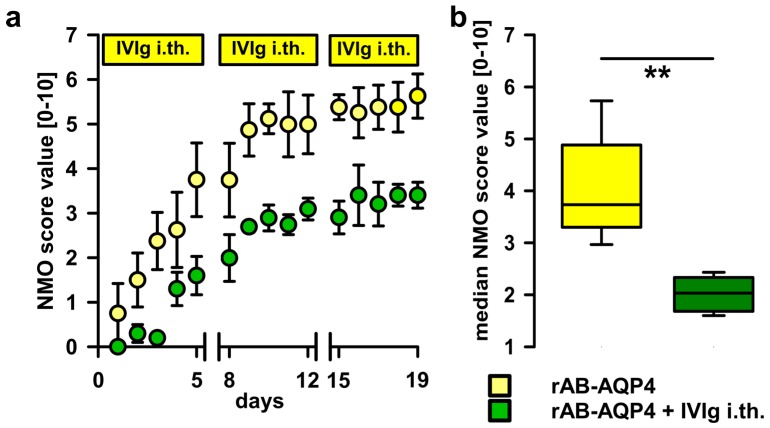
Effects of i.th. IVIg on disease progression induced by recombinant human anti-AQP4-ABs (rAB-AQP4). (**a**) i.th. rAB-AQP4 led to the development of moderate to severe disease signs (yellow circles) comparable to induction of disease using NMO-IgG. The signs of disease appeared later and the disease severity was reduced by concurrent i.th. injection of IVIg (Two-way ANOVA with Bonferroni post hoc test *p* < 0.001 for group comparison). Breaks on the X-axis indicate 2 day-pauses of NMO-IgG injections; (**b**) the treatment with i.th. IVIg over the entire experimental period led to a significant reduction of median NMO-Score (Mann-Whitney *U* test, ** *p* ≤ 0.01; plots show median ± 25th and 75th percentiles with whiskers of the 5th and 95th percentiles).

IVIg may act by a multitude of mechanisms, including direct interaction with pathogenic IgG ABs, e.g., by anti-idiotypic effects, steric hindrance, or increased catabolism. To test local effects on the ABs in the i.th. passive-transfer rat model, IVIg was co-administered by i.th. injection immediately after applying the pathogenic IgG fractions containing ABs to AQP4. When applying NMO-IgG 2 together with IVIg i.th., disease progression was delayed and disease scores were reduced (Figure 3). To validate AB-specificity of this finding, we used specific rAB-AQP4 as the pathogen in passive-transfer experiments. In line with previous studies, i.th. application of rAB-AQP4 led to induction of similar disease signs as NMO-IgG. Upon adding i.th. IVIg, we found similar beneficial effects on disease progression and severity, as seen with patient NMO-IgG (Figure 4).

## 3. Discussion

In AB-mediated autoimmune disorders of peripheral nerve and of the neuromuscular junction, systemic application of pooled human polyclonal immunoglobulin fractions (IVIg) has been proven as an effective treatment [17]. In NMOSD, only several case reports and case series were published that reported beneficial effects of IVIg as an interval treatment and as an acute treatment during relapses. In six patients IVIg given every two or three months reduced the annual relapse rate from 8.0 to 1.0 or to 0.75, respectively [13]. Other case series described similar observations with reduction of relapse rate and also clinical improvement [18,19]. Application of IVIg in the acute phase of NMOSD was also effective in a small retrospective case series of patients who did not respond to plasma exchange or steroid treatment [14]. 

In the present study, we demonstrate that systemic IVIg application was effective in reducing overall disease signs when studied in our passive-transfer animal model induced by i.th. application of ABs to AQP4 [10]. This model reflects only the intrinsic AB-driven pathology of NMOSD and, therefore, is suited to evaluate the role of polyclonal IVIg treatment. The disease-ameliorating effects were demonstrated independent of the mode of IVIg application with IgG ABs from two NMOSD patients. Equally effective was IVIg, when the experimental disease was induced by human recombinant ABs to AQP4. When IVIg was given before disease signs emerged, e.g., using a prophylactic paradigm, or when it was started at the height of the experimental myelopathy (therapeutic paradigm), the overall disability at the endpoint of 19 days was less pronounced than with no treatment and was not different between the two IVIg treatment paradigms. 

Systemically applied IVIg can have multiple effects on AB-dependent pathomechanisms, all of which may act in concert in autoimmune disorders, including NMODS. These mechanisms include increased IgG catabolism with clearance of pathogenic ABs, inhibition of complement activation, activation of B and T cells, suppression of AB production, and blockade of Fcγ receptors (for review see [16,20,21]. Addressing the question as to a possible direct blocking or neutralizing effect of IVIg, we applied IVIg and disease inducing NMO-IgG at the same site via a subarachnoid catheter. Indeed, disability was significantly less than without IVIg. This type of interaction was previously described in acute inflammatory neuropathy (Guillain-Barré Syndrome) [22] and in Lambert-Eaton myasthenic syndrome [23]. 

After intracerebral injection of NMO-IgG, Ratelade et al. reported a reduction of about 50% of demyelinating lesions when IVIg was applied by intraperitoneal injection [15]. In this study, the major effects of systemic IVIg was the inhibition of complement activation [15]. Since in our model neither complement activation nor cell mediated pathology is seen, we suggest that the treatment effects of systemic IVIg seen here are likely due to a direct interaction with pathogenic ABs. This is corroborated by showing equivalent effects by i.th. co-administration of pathogenic rAB-AQP4 and IVIg.

The mechanism of how IVIg interacts predominantly at the level of the pathogenic antibodies is not fully understood and could not be clarified by the present experiments in the living rat. In earlier ex vivo studies pathogenic IgG fractions from patients with Guillain-Barré Syndrome (GBS) [22] or Lambert Eaton Myasthenic Syndrome (LEMS) [23] could be neutralized by polyclonal IVIg. Dose dependent actions suggested direct competitive mechanisms at glycoconjugate antigen binding sites in the GBS experiments, presumably via steric hindrance phenomena [22]. In this study, the neutralizing effects were mediated by the Fab, but not by the Fc, part of IVIg [22]. It is important to note that, in our in vivo study, we cannot distinguish if IVIg effects are mediated by the Fab or Fc fragments of the polyclonal IgG molecules. In contrast to ex vivo studies, the amounts of Fab/F(ab)2 fragments needed for in vivo experiments in rat would be substantially higher than with native IgG (IVIg), which was not feasible in the current experiments. In our exclusively AB-mediated passive-transfer model, similar direct mechanisms of competitive inhibition [22] or anti-idiotypic effects [21] may apply for the interaction of IVIg and pathogenic ABs to AQP4, and this may explain the beneficial effects observed here. 

These findings complement those of Ratelade et al. [15] in providing experimental evidence that IVIg is effective in counteracting AB-induced intrinsic pathogenic effects. These experimental studies should not be regarded as preclinical studies to predict the effect of IVIg in patients, but they both may help elucidating the complex mechanisms of the beneficial action of IVIg in NMOSD. 

In clinical terms, large randomized, placebo-controlled cross-over clinical trials are warranted to validate the clinical efficacy of IVIg in NMOSD for relapse treatment and relapse prevention. 

## 4. Materials and Methods 

### 4.1. Patients, Therapeutic Plasma Exchange, and Preparation of IgG Fractions and rAB-AQP4

Purified NMO-IgG of two patients with NMOSD was prepared and used, as described in a previous study [10]. Briefly, purified IgG fractions were dialyzed against distilled water and stored at −20 °C after freeze drying. Lyophilized IgG was dissolved in normal saline just before use (concentration 100 mg/mL). Both patients fulfilled the revised diagnostic criteria for NMOSD [5] and had positive serum titers of ABs against AQP4 (titer ≥ 1:100). NMO-IgG and IgG and from a disease-control patient with chronic demyelinating inflammatory polyneuropathy that was negative for anti-AQP4 reactivity and negative for any antineuronal reactivity (control IgG) were purified from plasma filtrates, as described previously [10]. Generation and purification of rAB-AQP4 was described previously [8]. All patients declared consent for use of their IgG fractions. 

### 4.2. Animals, Surgery and Intrathecal Injections

A total of 45 rats were used for this study. All animals were purchased from Envigo (Horst, The Netherlands). All experiments were approved by the Bavarian and Thuringian State authorities and conducted according to the ARRIVE (Animal Research: Reporting of in vivo Experiments) guidelines [24]. Surgery and i.th. injections are described elsewhere [10,25]. i.th. catheters were placed, as described, and rats developing signs of paralysis after surgery were sacrificed immediately. After a recovery time of 8–10 days, rats were allocated to respective groups in a randomized manner and received i.th. and i.p. injections as indicated. As IVIg, we used commercial immunoglobulin preparations derived from about 60,000 healthy donors containing all IgG subclasses (Privigen^®^, CSL Behring, Marburg, Germany). i.p. injections of IVIg were applied in a dose of 40 mg/100 g body weight (IVIg concentration 100 mg/mL). In reference groups, rats received the same volume of saline, as reported in previous studies [26,27]. Systemic application of IVIg per se did not have any effects on motor function and coordination in rodents [27,28]. All i.th. injections were painless as judged by daily observation of the rats and were done in the awake rat while gently immobilizing them for 30 s. Ten microliters of NMO-IgG 1, 2 and control IgG, as well as IVIg, were injected at a concentration of 100 mg/mL in three series of five daily applications and a two-day break in between (Figure 1). rAB-AQP4 had about 100× higher specific activity, and was injected at a concentration of 1 mg/mL (10 µL per injection). Animals in the reference groups, i.e., without i.th. IVIg application, received an additional 10 µL of saline i.th.

### 4.3. Behavioral Analyses

Investigators who performed behavioral analyses were masked as to the treatment assignments. Body weight was monitored daily and animals were observed daily while moving in their cages and freely over a large table. Motor disability was rated on a spinal cord disease score modified from an Experimental Autoimmune Encephalomyelitis EAE score ranging from 0 (no symptoms) to 10 (death due to complete immobilization; Table 1) [10,29]. 

### 4.4. Data Analysis

All statistical analyses were performed with Sigma Plot 13.0 (Systat Software GmbH, Erkrat, Germany). Behavioral data were analyzed using a non-parametric Mann-Whitney *U* Test. Group comparisons were done using one- or two-way ANOVA with post hoc tests, as indicated in the figure legends. 

## 5. Conclusions

We examined the effect of IVIg in a rat model of NMOSD relying exclusively on the intrinsic pathogenic role of ABs to AQP4. Our results indicate that IVIg has a beneficial effect on the disease course when given in a prophylactic as well as in a therapeutic regime. The effects of IVIg in this animal model are likely mediated by direct antagonizing mechanisms with the pathogenic AQP4-ABs rather than interference with effector activation.

## Figures and Tables

**Table 1:** List of symptoms and respective scores. Animals were scored according to this table by investigators blinded to animal treatment on every day of autoantibody (AB) application before the actual injection.

Score	Symptoms
0	normal
1	reduced tone of tail
2	limp tail, impaired righting
3	absent righting
4	gait ataxia
5	mild paraparesis of hindlimb
6	moderate paraparesis
7	severe paraparesis or paraplegia
8	tetraparesis
9	moribund
10	death

## Abbreviations

AB	autoantibody
AQP4-AB	aquaporin-4 autoantibody
CNS	Central Nervous System
GBS	Guillain-Barré Syndrome
IVIg	pooled human immunoglobulins
NMO-IgG	purified immunoglobulin G from patients suffering from NMOSD
NMSOD	Neuromyelitis Optica Spectrum Disorder
i.p.	intraperitoneal
i.th.	intrathecal
rAB-AQP4	recombinant human anti-aquaporin-4 antibody
